# Rapid Screening of Phenolic Compounds with Anti-Enteritis Activity from *Camellia oleifera* Oil Using a Smurf Drosophila Model and Molecular Docking Methods

**DOI:** 10.3390/molecules29010076

**Published:** 2023-12-22

**Authors:** Shuhao Wang, Yang Li, Xin Lin, Xiangjin Fu, Haiyan Zhong, Kangzi Ren, Cheng Liu, Wen Yao

**Affiliations:** 1 Hunan Provincial Key Laboratory of Forest Edible Resources Safety and Processing Utilization, Central South University of Forestry and Technology, Changsha 410004, China; 2 Commodity Quality Inspection Institute of Hunan Province, Changsha 410004, China; 3 College of Food Science and Engineering, Central South University of Forestry and Technology, Changsha 410004, China; 4 Yi-Feng Agriculture and Forestry Technology Co., Ltd., Shaoyang 422300, China; 5 Hunan Ju Xiong Institute of *Camellia oleifera* Oil, Yueyang 414000, China; 19976978966@163.com

**Keywords:** *Camellia oleifera*, anti-enteritis activity, molecular docking, polyphenols, wighteone, *p*-octopamine

## Abstract

Screening and identifying the active compounds in foods are important for the development and utilization of functional foods. In this study, the anti-enteritis activity of ethanol extract from *Camellia oleifera* oil (*PECS*) was quickly evaluated using a Smurf Drosophila model and the metabolomics approach, combined with molecular docking techniques, were performed to rapidly screen and identify compounds with potential anti-enteritis activity in *PECS*. *PECS* showed good anti-enteritis activity and inhibited the activity of 5-lipoxygenase (LOX), cyclooxygenase 2 (COX-2) and inducible nitric oxide synthase (iNOS). In particular, wighteone and *p*-octopamine were newly identified in *C. oleifera* oil and were proven to have good anti-enteritis activity. The inhibitory activity of kaempferitrin (IC_50_ = 0.365 mmol L^−1^) was higher than that of wighteone (IC_50_ = 0.424 mmol L^−1^) and *p*-octopamine (IC_50_ = 0.402 mmol L^−1^). Of note, the IC_50_ value of salazosulfapyridine was 0.810 mmol L^−1^. Inhibition of LOX activity is likely one of the anti-enteritis mechanisms of *PECS*. These new findings lay the foundation for further investigations into the underlying mechanisms of anti-enteritis activity in *C. oleifera* oil.

## 1. Introduction

*Camellia oleifera* (*C. oleifera*) is a widely distributed plant in China and Southeast Asian countries; the oil processed from the seeds has high nutritional and medicinal values. The consumption of *C. oleifera* oil could reduce oxidative stress and inflammation [1,2], as well as confer gastrointestinal protection and anti-enteritis activity [3]. However, the active components and mechanisms are not fully understood.

Several studies have shown that polyphenols are key to the anti-inflammatory activity of *C. oleifera* oil. It has been reported that polyphenol extracts from *C. oleifera* oil could exert an anti-inflammatory effect by inhibiting the gene expression of nitric oxide synthase (iNOS) and cyclooxygenase 2 (COX-2), and by significantly inhibiting lipopolysaccharide -induced nitric oxide (NO) secretion [4]. Zhang et al. [5] evaluated the anti-inflammatory activity of phenolic compounds extracted from *C. oleifera* oil using a lipopolysaccharide-induced RAW 264.7 macrophage model, and identified 13 glycosylated polyphenols using ultra-high performance liquid chromatography with quadrupole time-of-flight mass spectrometry (UPLC-Q-TOF/MS); additionally, in this study, two of the glycosylated phenolic compounds extracted from *C. oleifera* oil were purified and proved to have anti-inflammatory activity. However, there are many other phenolic compounds which need to be identified. In additional, the existing methods for the identification of active polyphenols from foods require large amounts work of purification, which render them inefficient.

The efficiency of the identification of active polyphenols from foods can be significantly improved by combining metabolomics and molecular docking approaches. Metabolomics have the advantages of allowing for the simultaneous determination of a large number of metabolites in biological matrices, a comprehensive analysis of changes in metabolites’ content, and a reduced workload in the identification. Concurrently, molecular docking enables the prediction of the molecular recognition between ligands and enzymes [6], which allows for a rapid screening of highly active compounds, while reducing the workload in the purification. Rajesh et al. [7] used high-performance liquid chromatography (HPLC) and molecular docking to identify active compounds in the ethanolic extract of *Leucas vestita* Wall. ex. Benth, and explored the effect of these compounds on the activity of COX-2. In addition, Che et al. [8] employed molecular docking techniques to screen for potential anti-inflammatory active components in *Cybister chinensis*.

Another challenging aspect of the investigation of active compounds with anti-enteritis effects is that mice and rats are commonly used as models, which require long modeling times and the slaughtering of animals in order to determine the anti-inflammatory effects. Contrary to those using rodents, Drosophila has become a well-established and valuable model with high operational efficiency and low costs [9]. The mechanisms by which Drosophila can serve as a model for intestinal dysregulation and chronic inflammatory diseases have been discussed, and the immune signaling pathways of Drosophila share a high level of conservation with those of human beings [10]. In addition, anatomical similarities between the human and the Drosophila gut have been described, which supports employing Drosophila as a model to study diseases related to intestinal immunity dysfunction, including inflammatory bowel disease (IBD) [9]. Furthermore, Keshav et al. [11] showed that Drosophila treated with dextran sodium sulfate (DSS) led to an intestinal inflammatory response similar to that of IBD, thus being considered as a convenient model for studying IBD.

In the present study, the Smurf Drosophila model was used to rapidly determine the anti-enteritis activity of polyphenols extracted (70% ethanol was used as the extracting solvent) from *C. oleifera* oil (*PECS*), taking five famous plant polyphenol extracts (*PPE*) for comparing (cinnamon, CP; apple, AP; grape, GP; echinacea, EP; and pomegranate peel, PP). Moreover, a metabolomics approach based on ultra-high performance liquid chromatography with triple quadrupole linear ion trap mass spectrometer (UPLC-QTRAP/MS) was combined with molecular docking techniques to rapidly screen and identify polyphenol compounds with potential anti-enteritis activity in *PECS*.

## 2. Results and Discussion

### 2.1. Anti-Enteritis Activity of PECS

Inflammatory mediators, including prostaglandins, leukotrienes, NO, etc., play key roles in inflammation. In the arachidonic acid pathway, the inhibition of LOX and COX-2 activity reduces the production of prostaglandins and leukotrienes. Meanwhile, the inhibition of iNOS activity reduces the production of NO. These are two common mechanisms through which inflammation can be inhibited [4,12]. Thus, the inhibition rate of LOX, COX-2 and iNOS are usually used in evaluating the anti-inflammatory activity of bio-active compounds.

Moreover, an in-depth study of the mechanisms of intestinal inflammation requires in vivo models. In this context, the Smurf Drosophila model is considered less costly and more efficient for studying intestinal diseases compared to mammalian models. Herein, the Smurf Drosophila was chosen as the animal model used to study enteritis and intestinal damage [13]. In this assay, a blue dye which is not absorbed by the digestive tract is added to the feed. Since Drosophila possesses an open circulatory system, when intestinal integrity is disrupted due to enteritis, the blue dye enters the circulatory system and flies become blue in color, called “Smurf”. In contrast, when gut integrity is intact, the blue dye is found only in the gut and does not diffuse to other parts of the body. Thus, this method allows for quickly measuring intestinal permeability in Drosophila, as well as evaluating the anti-enteritis activity in samples [14].

**Figure 1:**
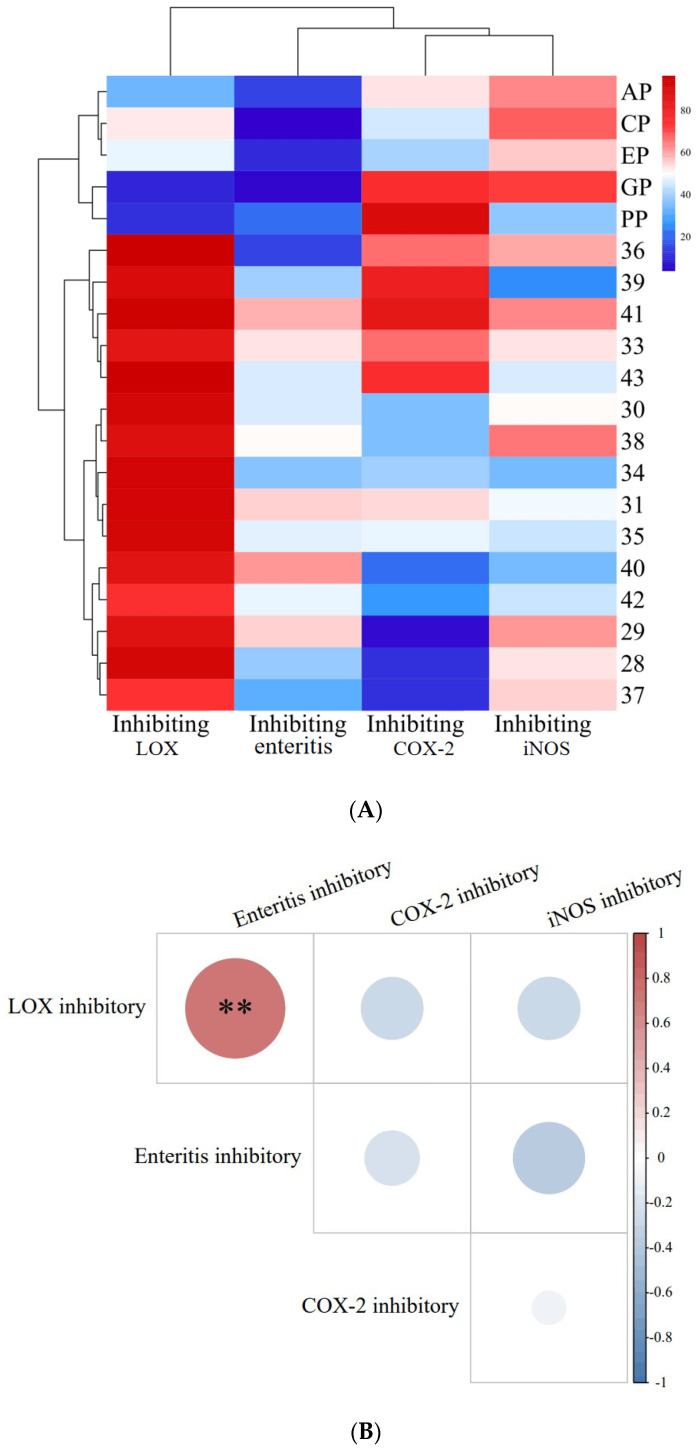
Inhibitory activity of *PECS* and *PPE* against enteritis, LOX, COX-2 and iNOS, and correlation analysis between these activities. In (**A**): The EP, CP, PP, AP and GP indicate echinacea polyphenol, cinnamon polyphenol, pomegranate peel polyphenol, apple polyphenol and grape skin polyphenol, respectively. In (**B**): the red dots indicate positive correlation, blue dots indicate negative correlation; the darker the color, the higher the correlation. ** indicates significant correlation at 0.01 level.

In the present study, the DSS was used to cause intestinal damage, like enteritis, in Drosophila. Figure 1A shows the inhibitory effect of *PECS* and *PPE* on enteritis and related enzymes (LOX, COX-2 and iNOS).

The inhibition rate of the medical positive control (salazosulfapyridine, PubChem CID: 5339) was 27.99 ± 2.00% (data shown in following Section 2.3), whereas the inhibition rates of *PECS* on enteritis were found to be within the range of 20–60%. The inhibition rate of *PPE* (cinnamon, CP; apple, AP; grape, GP; echinacea, EP; and pomegranate peel, PP) on enteritis in Drosophila was less than 20% and significantly lower (*p* < 0.05) than that of *PECS*. Therefore, *PECS* had a good inhibitory effect on intestinal inflammation, which is consistent with previous findings reported in the literature [3].

LOXs catalyze the reactions involved in the peroxidation of polyunsaturated fatty acids (arachidonic acid, linoleic acid, etc.). In addition, LOXs are involved in the production of inflammatory mediator leukotrienes (LTs) [12]. As shown in Figure 1A, all 20 extracts showed inhibitory activity against LOXs, with inhibition rates within the range of 10–95%. The inhibition rates of *PECS* were greater than 70%, and the inhibiting rates of samples No. 28, 30, 31, 34, 35, 36, 39, 41 and 43 were greater than 90%. In contrast, the inhibition rates of *PPE* were less than 60%.

COX-2 catalyzes the production of prostaglandin E2 (PGE2) from arachidonic acid [15]. PGE2 is one of the most potent mediators of inflammation, being involved in all inflammatory processes, and leading to typical signs of inflammation such as pain, swelling and redness [16]. Therefore, COX-2 is an important target for inhibiting inflammation. The currently available anti-inflammatory drugs (e.g., ibuprofen and aspirin) are synthetic COX-2 inhibitors whose anti-inflammatory effects rely on blocking the COX-2 pathway during inflammation [17]. As shown in Figure 1A, all 20 extracts (*PECS* and *PPE*) evaluated herein showed inhibitory activity against COX-2, with inhibition rates within the range of 10–90%; in particular, samples No. 39, 41 and PP (pomegranate peel extract) could inhibit COX-2 by more than 80%, which was significantly higher than other samples (*p* < 0.05).

Inducible iNOS is a key enzyme involved in NO production. Excessive NO production can cause tissue damage and is involved in the pathogenesis of several acute and chronic inflammatory diseases. Therefore, inhibiting iNOS activity is important for halting inflammation. As shown in Figure 1A, all 20 extracts (*PECS* and *PPE*) showed inhibiting effect on iNOS activity, with inhibition rates ranging from 25 to 70%; more specifically, samples No. 29, 36, 38, 40, CP (cinnamon polyphenol extract), AP (apple polyphenol extract) and GP (grape skin polyphenol extract) showed inhibition rates greater than 60%.

Based on the above data, *PECS* showed good anti-enteritis activity, most likely by regulating inflammatory factors as a result of the inhibition of LOX, COX-2 and iNOS activity, thereby showing anti-inflammatory effects, which is consistent with the findings reported by [3]. In addition, the anti-enteritis effect of *PECS* was superior to that of *PPE*, i.e., cinnamon polyphenol (CP); apple polyphenol (AP); grape skin polyphenol (GP); echinacea polyphenol (EP); and pomegranate peel polyphenol (PP).

An analysis of the correlation between the above indexes’ activity on *PECS* is shown in Figure 1B. There is a highly significant positive correlation (*p* < 0.01) between the LOX inhibitory activity and anti-enteritis activity in Smurf Drosophila model. This observation is consistent with the traditional theory about the association between LOX activity and inflammation, and the inhibition of LOX activity might be one of the anti-enteritis mechanisms of *PECS*.

### 2.2. Screening of Phenolic Compounds in PECS with Anti-Enteritis Activity

#### 2.2.1. Metabolomics Analysis Based on UPLC-QTRAP/MS

**Figure 2:**
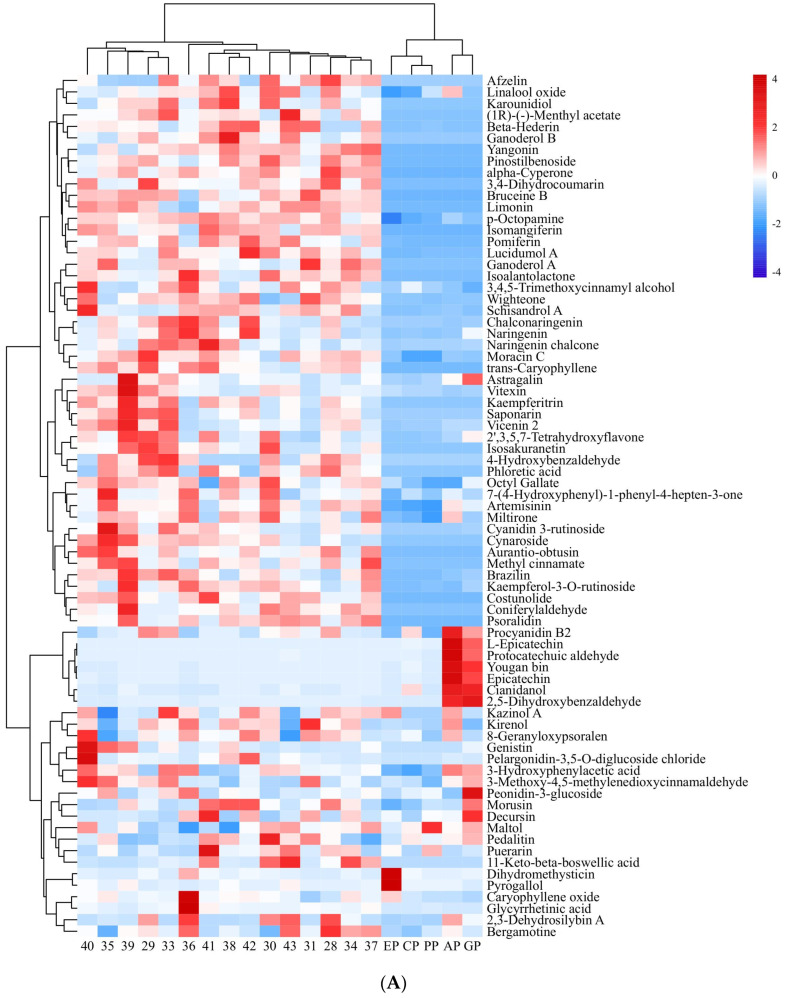

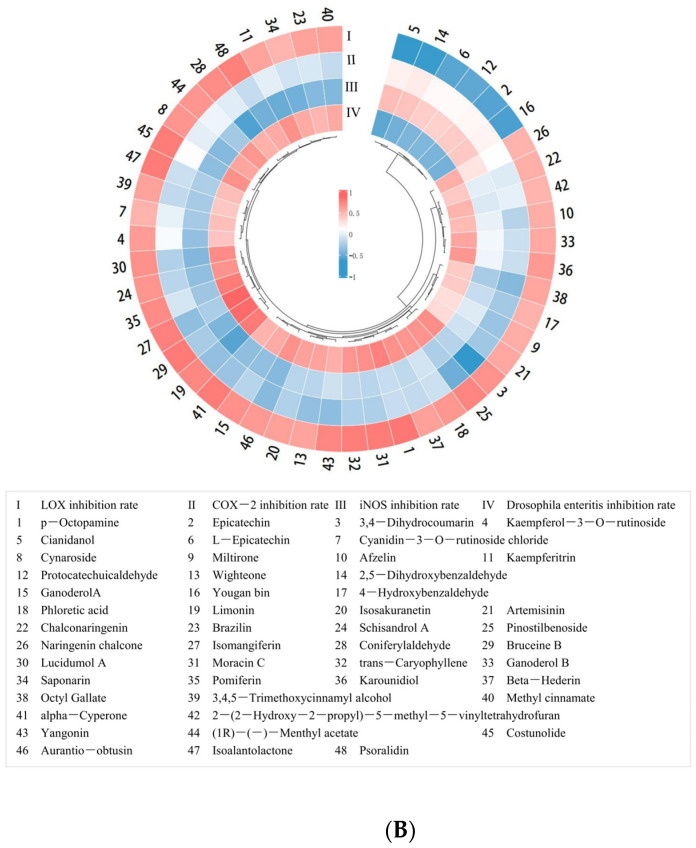
Heatmap of small-molecule organic compounds’ content and correlation coefficient. (**A**): Heatmap of small-molecule organic compounds’ content. The numbers indicate samples of *PECS*. The EP, CP, PP, AP and GP indicate echinacea polyphenol, cinnamon polyphenol, pomegranate peel polyphenol, apple polyphenol and grape skin polyphenol, respectively. Red indicates high content; blue indicates low content. (**B**): Heatmap of correlation coefficient between small-molecule organic compounds’ content and the anti-enteritis activity of ethanol extracts (*PECS* and *PPE*). The numbers indicate the small-molecule organic compounds. Red indicates positive correlation; blue indicates negative correlation; and the darker the color, the higher the correlation.

Small-molecule organic compounds in 15 *PECS* and five *PPE* (ethanol extracts from cinnamon, apple, grape skin, echinacea and pomegranate peel) were detected and identified by UPLC-QTRAP/MS. In total, 885 peaks were retained after pretreatment of raw data. Among these, polyphenolic compounds were the most abundant. A total of 75 small-molecule organic compounds whose relative contents were greater than 10 were considered for further analysis, and their contents are shown in Figure 2A.

Polyphenols can be classified, based on their structures, into flavonoids, phenolic acids, lignans and coumarins [17,18]. Among polyphenolic metabolites found in *PECS*, flavonoids were the most abundant, followed by phenolic acids, with few lignans and coumarins. In total, 32 polyphenolic compounds whose relative content was above 100 were found, which included 18 flavonoids, such as 2,3-dehydrosilybin A, 2′,3,5,7-tetrahydroxyflavone, astragalin, among others; 6 phenolic acid compounds, including 2,5-dihydroxybenzaldehyde, 4-hydroxybenzaldehyde and *p*-octopamine (PubChem CID: 440266), among others; one lignan (schisandrol A); and one coumarin (8-geranyloxypsoralen).

In a previous study, Wang et al. [19] evaluated *C. oleifera* oil obtained from 15 regions of China and identified 24 phenolic compounds, including cynaroside, epicatechin (PubChem CID: 72276), kaempferitrin (PubChem CID: 5486199) and kaempferol-3-*O*-rutinoside (PubChem CID: 5318767). In addition, Hong et al. [20] evaluated methanolic extracts fro, three *C. oleifera* seed cakes and identified 73 phenolic compounds, including four flavonoids, among which were included cynaroside, epicatechin, kaempferitrin, kaempferol-3-*O*-rutinoside and procyanidin B2. Herein, the phenolic compounds afzelin, cyanidin 3-rutinoside, wighteone, *p*-octopamine, miltirone and 8-geranyloxypsoralen were identified for the first time in *C. oleifera* oil. Afzelin is a phenolic compound found, e.g., in *Pithecellobium dulce*, and shows good antioxidant, anti-inflammatory [21] and anti-tumor [22] activities. Cyanidin 3-rutinoside is a natural anthocyanin found in a variety of plants such as mulberry, cherry and black tree plum, which shows good antioxidant, anti-inflammatory [23] and hypoglycemic [24] activities. Wighteone (PubChem CID: 5281814) is a natural flavonoid isolated from the roots of *Erythrina variegata* Linn. and *Pueraria lobata*, and whose antitumor [25] and antibacterial [26] activities have been reported, but the anti-inflammatory activity has not yet been reported. *p*-octopamine is commonly found in plants, invertebrates and animals. It reached a concentration of 16.3 mg mL^−1^ in the juice of Meyer lemons [27] and has remarkable pharmacological and physiological effects such as regulating human metabolism and promoting lipolysis [28], with good prospects for application in the prevention and treatment of obesity and type II diabetes; however, its anti-inflammatory activity has not yet been reported.

#### 2.2.2. Correlation Analysis between Small-Molecule Organic Compounds’ Content and Anti-Enteritis Activity of *PECS*

Furthermore, Pearson correlation analysis was conducted with data on the content of 75 small-molecule organic compounds in *PECS* and the anti-enteritis activity of *PECS*. As shown in Figure 2B, 42 small-molecule organic compounds showed significant positive correlation with LOX inhibitory activity, whereas seven compounds showed significant negative correlation (*p* < 0.05). No compounds showed a significant positive correlation with COX-2 inhibitory activity. Cianidanol was the only compound which showed a significant positive correlation with iNOS inhibitory activity (*p* < 0.05).

Furthermore, 35 small-molecule organic compounds showed a significant positive correlation (*p* < 0.05) with enteritis inhibitory activity in Drosophila, while three compounds showed a significant negative correlation with enteritis inhibitory activity (*p* < 0.05). Among the compounds with a significant positive correlation with enteritis inhibitory activity, nine compounds were found to have a relative content greater than 100, i.e., cyanidin 3-rutinoside, cynaroside, afzelin, isomangiferin, kaempferitrin, naringenin chalcone, kaempferol-3-*O*-rutinoside, limonin (PubChem CID: 179651), bruceine B. Moreover, nine compounds which have not been reported to have anti-inflammatory activity, namely *p*-octopamine, phloretic acid, pinostilbenoside, (1*R*)-(−)-menthyl acetate, ganoderol B, karounidiol, wighteone, lucidumol A, beta-hederin, were selected for molecular docking analysis.

#### 2.2.3. Molecular Docking Analysis

Table 1 shows the minimum docking binding energy and RMSD of predicted docked conformations between the 18 potential active compounds and LOX, COX-2 and iNOS. The lower the docking binding energy, the more stable the conformation and the more likely it is to lead to enzyme inhibition [29]. RMSD allows for assessing the accuracy of the docked conformation between the ligand and receptor. The lower the RMSD value, the closer the ligand and the receptor [30]. It is generally considered that a docking binding energy below −5 kcal mol^−1^ indicates the docking conformation is stable, and RMSD values below 2 Å indicate that the ligand and the receptor are effectively docked [31]. As shown in Table 1, ganoderol B, karounidiol, wighteone, lucidumol A, β-hederin, limonin, bruceine B, kaempferitrin and kaempferol-3-*O*-rutinoside showed stable conformation when docked with LOX, COX-2 and iNOS. They are polyphenols and terpenoids.

### 2.3. Screening of Polyphenolic Compounds in PECS with Anti-Enteritis Activity Using the Smurf Drosophila Assay

**Figure 3:**
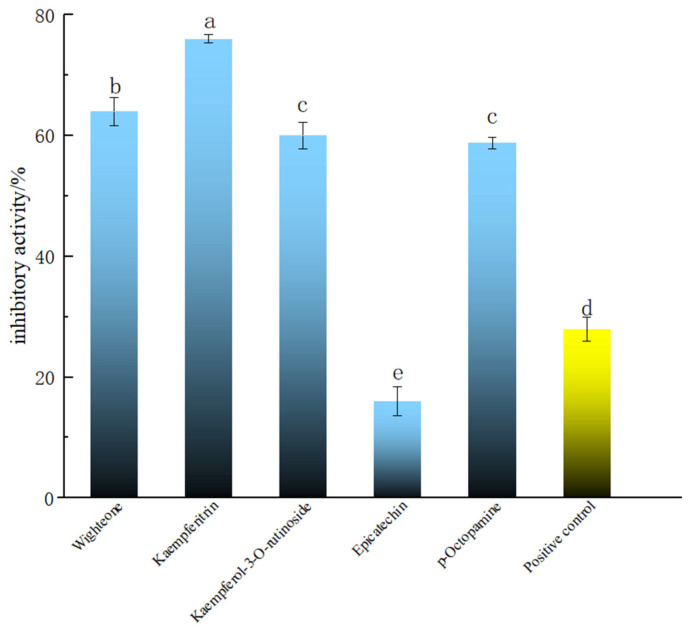
Anti-enteritis activity of different polyphenolic compounds in Smurf Drosophila model. Blue bars represent polyphenolic compounds; the yellow bar represents the medical positive control (salazosulfapyridine). a–e: Mean inhibitory activities with different letters are significantly different (*p* < 0.05) (n = 6).

The compounds with good molecular docking results and a relative content >500 (i.e., wighteone, kaempferitrin and kaempferol-3-*O*-rutinoside), and the newly found phenolic compound in *PECS* with relative content >500 (*p*-octopamine), were tested in the Smurf assay in Drosophila. Epicatechin is one of the main components of *PECS*, whose anti-inflammatory activity has been previously reported [2]. Thus, for the purposes of comparison, epicatechin was used as the reference and salazosulfapyridine as the positive control in the Smurf assay experiments. The results are shown in Figure 3. It was found that wighteone and kaempferitrin showed inhibition rates greater than 60%, which were significantly higher than those of epicatechin and salazosulfapyridine (27.99 ± 2.00%) (*p* < 0.05). Kaempferitrin is found in *C. oleifera* and has demonstrated anti-inflammatory activity [32]; herein, kaempferitrin docked well with LOX, COX-2 and iNOS (Table 1) and had high enteritis inhibitory activity in Drosophila (Figure 3). In addition, wighteone and *p*-octopamine are phenolic compounds newly detected in *C. oleifera* oil in present study, and whose anti-inflammatory functions have not been previously reported. Therefore, the anti-enteritis activity of kaempferitrin, wighteone and *p*-octopamine were further investigated.

### 2.4. Inhibition of COX-2, LOX, and iNOS Activity by Phenolic Compounds

**Figure 4:**
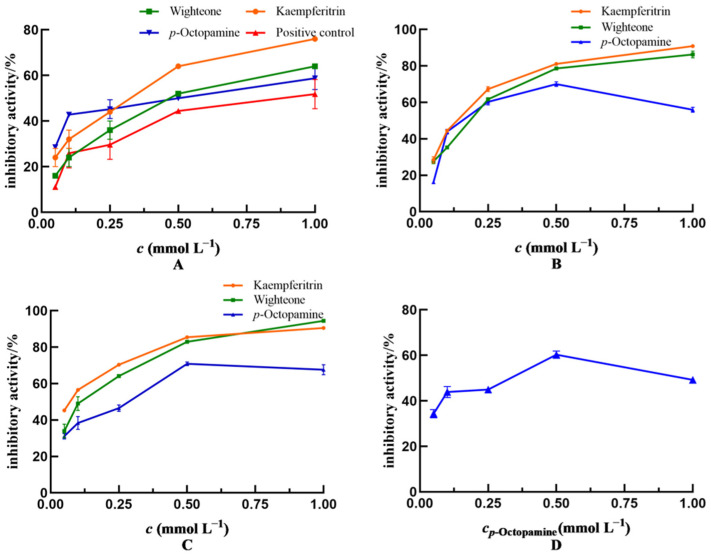
Anti-enteritis activity of kaempferitrin, wighteone and *p*-octopamine. (**A**): Inhibiting enteritis in Smurf Drosophila model. Positive control: salazosulfapyridine. (**B**–**D**): Inhibiting the activity of lipoxygenase (LOX), cyclooxygenase (COX-2) and inducible nitric oxide synthase (iNOS), respectively. In (**D**), only *p*-octopamine showed inhibiting activity against iNOS.

Subsequently, the capacity of kaempferitrin, wighteone and *p*-octopamine to inhibit enteritis in Drosophila was evaluated, and the results are shown in Figure 4A. The inhibitory effects on enteritis in Drosophila increased gradually with increasing concentrations of these polyphenolic compounds, indicating that the inhibitory activity was dose dependent. The inhibitory activity of kaempferitrin (IC_50_ = 0.365 mmol L^−1^) was higher than that of wighteone (IC_50_ = 0.424 mmol L^−1^) and *p*-octopamine (IC_50_ = 0.402 mmol L^−1^). Of note, the IC_50_ value of salazosulfapyridine was 0.810 mmol L^−1^. The kaempferitrin, wighteone and *p*-octopamine showed greater enteritis inhibitory activity in Drosophila, compared with the drug positive control (salazosulfapyridine).

The inhibitory effects of kaempferitrin, wighteone and *p*-octopamine on LOX activity is shown in Figure 4B. The inhibitory activity of the three tested compounds on LOX was dose dependent. In particular, the inhibitory effect of kaempferitrin (IC_50_ = 0.130 mmol L^−1^) was stronger than that of *p*-octopamine (IC_50_ = 0.177 mmol L^−1^) and wighteone (IC_50_ = 0.216 mmol L^−1^).

Figure 4C depicts the in vitro inhibitory effects of kaempferitrin, wighteone and *p*-octopamine on COX-2 activity. Their inhibitory effects on COX-2 were dose dependent. In addition, the inhibitory effect of kaempferitrin (IC_50_ = 0.186 mmol L^−1^) was greater than that of wighteone (IC_50_ = 0.279 mmol L^−1^) and *p*-octopamine (IC_50_ = 0.214 mmol L^−1^).

Figure 4D shows the inhibitory effect of *p*-octopamine on iNOS activity at an IC_50_ of 0.241 mmol L^−1^. Kaempferitrin and wighteone showed no inhibitory effect on iNOS activity, which is inconsistent with the molecular docking results (Table 1); this is likely due to the fact that the active site in iNOS consists of an elongated active lumen and a circular active pocket, hence kaempferitrin and wighteone could not reach the circular active pocket to establish hydrogen bonds when mimicking binding to the iNOS.

## 3. Materials and Methods

### 3.1. Materials

*C. oleifera* oil was provided by Dongkou Yifeng Agriculture and Forestry Co., Ltd. (Shaoyang, China). In total, 15 samples of *C. oleifera* oil (numbered 28, 29, 30, 31, 33~43) were collected from September to December 2020 in Dongkou County, Shaoyang City, Hunan Province, China.

We used five plant polyphenol extracts (*PPE*) for comparison: cinnamon polyphenol (CP), apple polyphenol (AP), grape skin polyphenol (GP), echinacea polyphenol (EP) and pomegranate peel polyphenol (PP); they were purchased from Shanxi Hengling nature bio-products Ltd. Com. (Xi’an, China).

Phenolic compounds (wighteone, kaempferitrin, kaempferol-3-*O*-rutinoside, limonin, *p*-octopamine and epicatechin) were purchased from Yuanye biotec. Ltd. Com. (Shanghai, China). The dextran sodium sulfate (DSS) and salazosulfapyridine were purchased from A-la-ding biochemical tec. Ltd. Com. (Shanghai, China).

Wild-type *Drosophila melanogaster* was cultured in a thermostat at 25 ± 1 °C. The formula of feed was as follows: yeast powder 24.50 g, agar 10.00 g, sucrose 7.25 g, brown sugar 30 g, corn flour 50.00 g, dissolved in 500 mL water followed by boiling for 20 min, and then left to cool naturally for 5min, and finally 4.0 mL of propionic acid was added [32].

### 3.2. Preparation of Polyphenol Extracts from C. oleifera Oil

*C. oleifera* oil was mixed with 70% ethanol solution at a ratio of 1:15 (*v*/*v*), and extraction was carried out at 60 °C, at an ultrasonic power of 100 W, for 30 min. Then, the mixture was submitted to centrifugation at 2000× *g* for 15 min, and the supernatant was obtained and evaporated at 60 °C in a rotary evaporator (being considered polyphenol extracts from *C. oleifera* oil, *PECS*), freeze-dried and stored at 4 °C [33].

### 3.3. Determination of Anti-Inflammatory Activity In Vivo

The anti-inflammatory activity of *PECS*, *PPE* and phenolic compounds (dissolved in ethanol, 0.1 mg mL^−1^) were determined in Smurf Drosophila model based on the method proposed by Yu et al. [34], using salazosulfapyridine (dissolved in ethanol, 0.1 mg mL^−1^) as a medical positive control.

We added 5% DSS and 2.5% Brilliant Blue R into the model group’s feed; 5% DSS, 2.5% Brilliant Blue R and samples (0.1 mg mL^−1^) were added into the experimental group’s feed. After 12 h of feeding, stained Drosophila were observed under a microscope (×20); a blue color in the abdominal cavity and thorax of Drosophila indicated intestinal leakage caused by enteritis. The inhibition rate for enteritis in Drosophila was calculated based on the following formula:

$$C_{{}_{1}}=(\frac{\;“\mathrm{Smurf}”\;\mathrm{Number}\;\mathrm{in}\;\mathrm{Model}\;\mathrm{Group}-“\mathrm{Smurf}”\;\mathrm{Number}\;\mathrm{in}\;\mathrm{Sample}\;\mathrm{Group}}{\;“\mathrm{Smurf}”\;\mathrm{Number}\;\mathrm{in}\;\mathrm{Model}\;\mathrm{Group}})\times 100\;$$

where *C*_1_ is the inhibition rate for enteritis in Drosophila (%).

### 3.4. Determination of the Inhibitory of 5-Lipoxygenase (LOX), COX-2, and iNOS

The inhibition of LOX activity was determined based on a method previously described [35], and linoleic acid was used as the substrate.

The inhibition of COX-2 activity was determined using a cyclooxygenase inhibition screening kit (Beyotime Biotechnology, Shanghai, China).

The inhibition of iNOS activity was determined using the iNOS assay kit (Nanjing Jiancheng Bioengineering Institute, Nanjing, China).

### 3.5. Metabolomics Analysis Based on UPLC-QTRAP/MS

An aliquot (20 mg) of sample was weighed and transferred to an Eppendorf tube, and 500 μL of extract solution (methanol/water, 3:1, *v*/*v*; precooled at −40 °C) was added, followed by vortexing for 30 s. Then, samples were homogenized at 35 Hz for 4 min and sonicated for 5 min in ice-water bath. The homogenization and sonication steps were repeated three times. Samples were extracted overnight at 4 °C in a shaker at 100 r min^−1^, then submitted to centrifugation at 13,800× *g* for 15 min at 4 °C (Heraeus Fresco17, Thermo, Waltham, MA, USA). The supernatant was carefully filtered through a 0.22 μm filter membrane, and the resulting filtrates were diluted 50 times in a methanol/water solution (3:1, *v*/*v*) and vortexed for 30s, then transferred to 2 mL glass vials. An aliquot (40 μL) of each sample was obtained and pooled as quality control samples. Samples were stored at −80 °C until UPLC-QTRAP/MS (Sciex, Framingham, MA, USA) analysis.

Target compounds were separated chromatographically in an EXION LC ultra-high performance liquid chromatograph (Sciex, Framingham, MA, USA) with a UPLC liquid chromatography column (Waters, Milford, MA, USA). The mobile phase A was 0.1% formic acid in water, and the mobile phase B was acetonitrile With a flow rate of 400 μL min^−1^; gradient elution program: 0–0.5 min, 98% A, 2% B; 0.5–11 min, 5–98% A, 2–95% B; 11–13 min, 5% A, 95% B; 13–13.1 min, 5–98% A, 2–95% B; 13.1–15 min, 98% A, 2% B; column temperature 40 °C; injector temperature 4 °C; injection volume 2 μL.

Mass spectrometry was performed in multiple reaction monitoring (MRM) mode using a 6500 QTRAP mass spectrometer equipped with an IonDrive Turbo V ESI ion source (Sciex, Framingham, MA, USA). Typical ion source parameters were: IonSpray Voltage, +5500/−4500 V; curtain gas, 35 psi; temperature, 400 °C; ion source gas, 1:60 psi; ion source gas, 2:60 psi; DP, ±100 V.

### 3.6. Molecular Docking Analysis

Protein crystal structures of LOX (PDB ID: 1YGE), COX-2 (PDB ID: 5F19) and iNOS (PDB ID: 2NSI) were downloaded from the PDB database (http://www.rcsb.org, accesssed on 30 July 2022) and saved in PDB format for docking. Protein crystal structures were processed using Autodock Tools 1.5.7 software (Olson, Orange City, FL, USA) to remove solvent molecules, etc., and saved as PDBQT format. Structures of ligands (polyphenonic compound) were downloaded from the ZINC (https://zinc.docking.org, accessed on 30 July 2022) and the PubChem (https://pubchem.ncbi.nlm.nih.gov, accessed on 30 July 2022), and were processed for hydrogenation and charge calculation using Autodock Tools (Arthur Olson, The Center for Computational Structural Biology). Binding of ligand to the receptor (LOX, COX-2, and iNOS) was predicted using Autodock Tools. The binding between ligand and the receptor was determined using a semi-empirical free energy calculation method based on a Lamarckian genetic algorithm.

### 3.7. Statistical Analysis

The acquisition and processing of mass spectrometry data were performed with SCIEX Analyst Work Station 1.6.3 (Sciex, Framingham, MA, USA). Raw mass spectra were converted into .txt format using MS converter 3.0.19059-2449a7a08 software, an open-source, cross-platform software provided by ProteoWizard 3.0.21229 that facilitates metabolomics raw data format conversion. Peak extraction and annotation were then performed using a self-authored R package in combination with a self-built database.

All anti-inflammatory activity assays were repeated at least three times, and the results reported are the average of obtained measurements. The SPSS 22.0 software (IBM Corporation, Armonk, NY, USA) was used to correlate anti-inflammatory activity with metabolomics data. GraphPad Prim 8 (GraphPad Software Inc., San Diego, CA, USA) was used to draw figures.

## 4. Conclusions

Herein, a method for the rapid screening of active compounds in *C. oleifera* oil was developed. The novel method proposed enables a reduction in the costs of isolating and purifying the active compounds. The trend of anti-enteritis activity of *PECS* was consistent with the trend of the inhibition of LOX activity, which suggests that the anti-enteritis activity of *PECS* might be mainly attributed to the inhibition of LOX activity by its polyphenolic components, which is a new finding from the present study. More importantly, two new polyphenolic compounds were identified in *C. oleifera* oil, i.e., wighteone and *p*-octopamine, whose anti-inflammatory activity was described for the first time. Thus, the findings of the present study lay the foundation for further investigations into the anti-enteritis mechanism of *C. oleifera* oil.

## Figures and Tables

**Table 1:** Root mean square deviation (RMSD) and docking binding energy values of docking conformations between small-molecule organic compounds with lipoxygenase (LOX), cyclooxygenase (COX-2) and inducible nitric oxide synthase (iNOS).

Phenolic Compounds	Docking Binding Energy (kcal mol^−1^)			RMSD		
	LOX	COX-2	iNOS	LOX	COX-2	iNOS
*p*-Octopamine	−5.1	−5.31	−3.3	1.760	1.770	1.731
3-(2-hydroxyphenyl)propanoic acid	−4.82	−4.05	−4.75	2.522	2.638	2.677
cyanidin 3-rutinoside	−5.77	−5.20	−3.26	2.772	1.974	1.987
(1*R*)-(−)-Menthyl acetate	−5.25	−5.04	−4.8	0.000	0.000	0.000
Ganoderol B	−9.47	−11.16	−9.59	0.001	0.527	0.001
Karounidiol	−12.23	−12.11	−10.7	0.000	0.000	0.000
Wighteone	−5.56	−5.56	−5.03	0.000	0.229	0.001
Lucidumol A	−10.06	−12.83	−10.83	0.000	0.000	0.294
β-Hederin	−8.68	−13.62	−11.41	0.624	0.767	0.521
Limonin	−6.40	−7.04	−6.33	0.000	0.000	0.000
Isomangiferin	−2.96	−4.41	−4.92	0.001	0.008	0.001
Chalconaringenin	−2.17	−0.84	−2.09	1.918	0.431	1.004
Bruceine B	−6.25	−5.87	−5.62	0.001	0.001	0.000
Afzelin	−3.62	−3.54	−3.15	0.484	0.522	0.601
Cynaroside	−3.60	−6.00	−3.04	1.335	0.784	1.318
Kaempferitrin	−9.27	−13.05	−9.51	0.323	0.352	0.588
Kaempferol-3-*O*-rutinoside	−10.49	−14.49	−10.62	0.596	0.650	0.460
Cyanidin 3-rutinoside	−5.77	−5.20	−3.26	2.772	1.974	1.987

## Data Availability

Data are contained within the article.
